# Ceruminous Adenoma of the External Auditory Canal: A Case Report with Imaging and Pathologic Findings

**DOI:** 10.1155/2015/359627

**Published:** 2015-11-17

**Authors:** George Psillas, Argyrios Krommydas, Georgia Karayannopoulou, Kyriakos Chatzopoulos, Jean Kanitakis, Konstantinos Markou

**Affiliations:** ^1^1st Academic ENT Department, Aristotle University of Thessaloniki, AHEPA Hospital, 1 Stilponos Kyriakidi Street, 546 36 Thessaloniki, Greece; ^2^Department of Pathology, Aristotle University of Thessaloniki, School of Medicine, 541 24 Thessaloniki, Greece; ^3^Department of Dermatology, Ed. Herriot Hospital Group, Lyon, France

## Abstract

Ceruminous adenomas are benign tumors that are rare in humans and present with a nonspecific symptomatology. The treatment of choice is surgical excision. We present an 87-year-old woman who presented with a reddish, tender, round, soft mass of the outer third of the inferior wall of the left external auditory canal, discharging a yellowish fluid upon pressure. Coincidentally, due to her poor general condition, this patient also showed symptoms consistent with chronic otitis media, parotitis, and cervical lymphadenopathy, such as otorrhea, through a ruptured tympanic membrane and swelling of the parotid gland and cervical lymph nodes. The external auditory canal lesion was surgically excised under general anesthesia, utilizing a transmeatal approach. The pathological diagnosis was ceruminous gland adenoma. The tumor was made of tubular and cystic structures and embedded in a fibrous, focally hyalinized stroma. Immunohistochemistry confirmed the presence of two distinct cell populations. The luminal cells expressed keratin 7, while peripheral (basal) cells expressed keratins 5/6, S100 protein, and p63. The apocrine gland-related antigen GCDFP-15 was focally expressed by tumor cells. The postoperative course was uneventful and at the 2-year follow-up no recurrence of the ceruminous adenoma was noted.

## 1. Introduction

Ceruminous adenoma (CA) is a benign neoplasm of ceruminous glands, developing exclusively in the external auditory canal (EAC) [1]. Contrary to felines and canidae [1–8], it is rare in humans and poses a diagnostic problem for the clinician, due to the variety of clinical presentations. The wavering nomenclature existing for these lesions causes additional confusion to pathologists and attending doctors [2]. We present herein a new case of CA and briefly review the relevant literature in order to delineate the salient clinicopathological features of this rare tumor [9].

## 2. Case Report

An 87-year-old woman was referred to our department for otalgia, yellowish discharge, itching, and hearing loss of the left ear recurring over the last three months. She reported intermittent fever, but neither tinnitus nor vertigo. She denied previous trauma or surgery in this region and was not using a hearing aid. Her past history included atrial fibrillation, diabetes mellitus, arterial hypertension, and glaucoma. Oral antibiotics and anti-inflammatory medications, prescribed elsewhere, were not effective.

**Figure 1:**
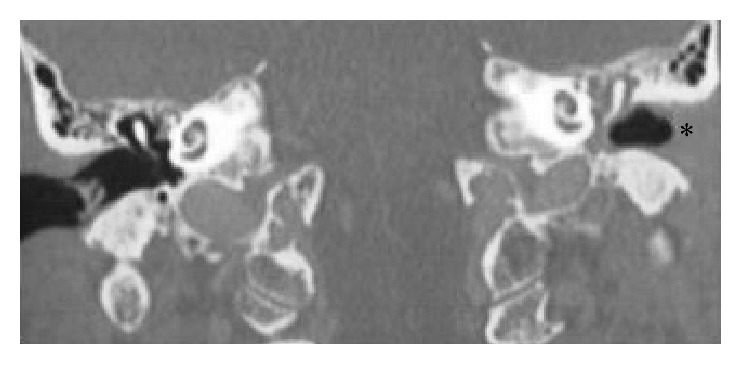
CT imaging depicting otitis media of the left ear with external ear canal obstruction (*∗*) due to the cystic lesion.

Physical examination revealed a reddish, round, tender soft mass of the outer third of the inferior wall of the left EAC, discharging a yellowish fluid upon pressure; however, a concomitant ruptured tympanic membrane and otorrhea through this perforation were also found. No cranial nerve palsy was detected. Swelling of the left parotid region and bilateral cervical lymphadenopathy were also present. CT scan of the temporal bone showed a cystic lesion on the inferior cartilaginous part of the EAC but no temporal bone lysis; the middle ear appeared to contain fluid and swelling of the parotid gland and cervical lymph nodes bilaterally were also seen (Figure 1). The audiogram revealed conductive hearing loss of the left ear. The patient was treated with intravenous cefuroxime. A specimen of the discharge was sent for bacteriological evaluation and turned out to be negative for bacteria.

The patient underwent excision of one cervical lymph node and fine needle aspiration (FNA) of the left parotid. Histopathological examination of the lymph node ruled out malignancy, being consistent with a granulomatous process; however blood tests and thorough immunological and rheumatological examination failed to confirm this hypothesis. The FNA examination was nondiagnostic as it showed normal salivary glands.

**Figure 2:**
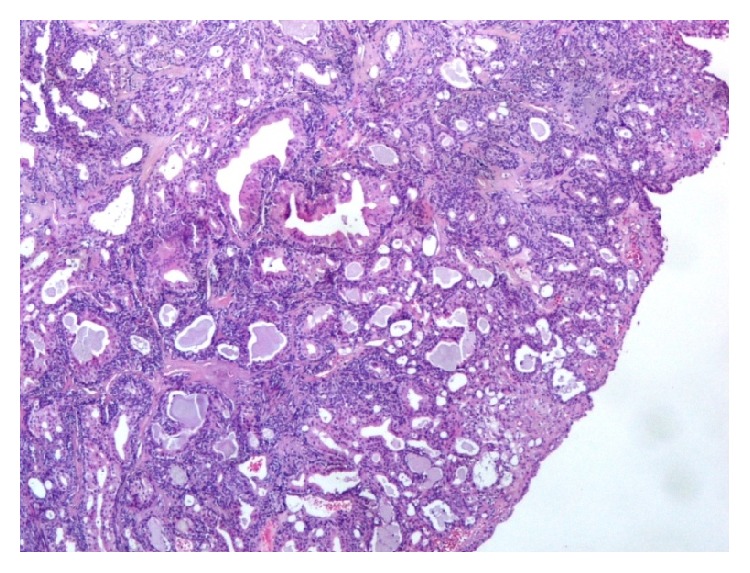
H/E ×40. The tumor is made of tubular and cystic structures.

**Figure 3:**
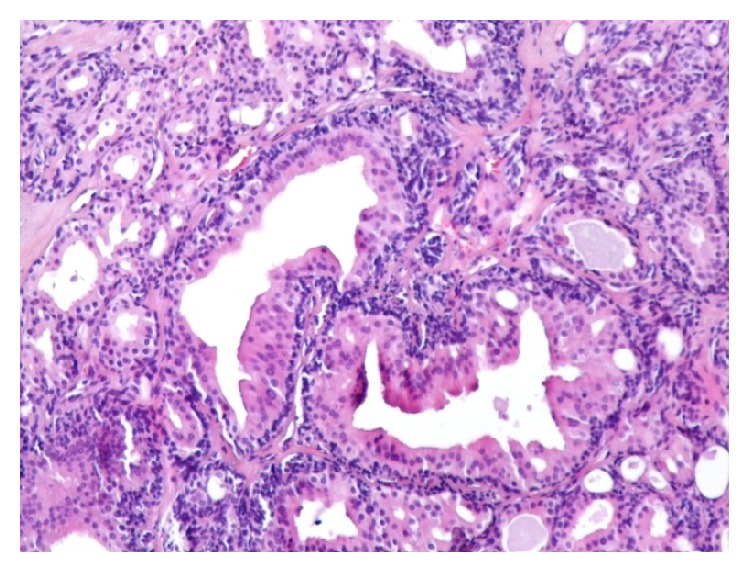
H/E ×100. Two cystic-tubular formations lined by columnar cells with an eosinophilic cytoplasm are seen.

**Figure 4 fig4:**
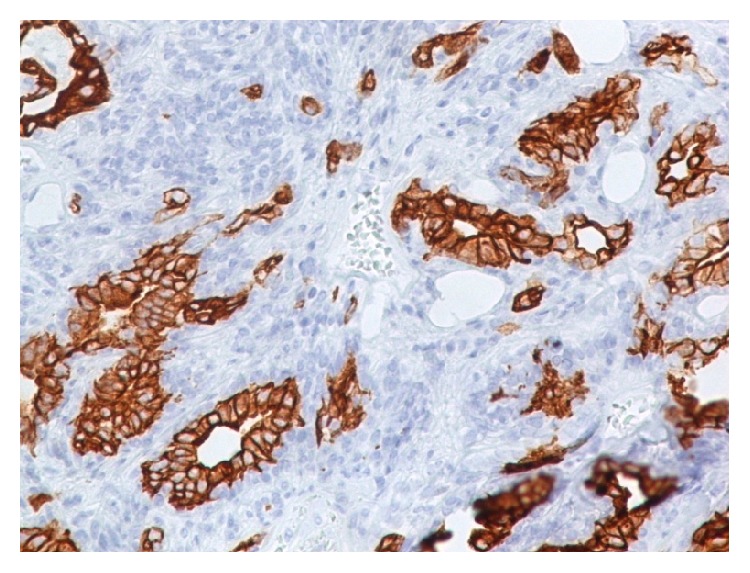
Immunostain for keratin 7 (×100): luminal cells are labelled.

**Figure 5:**
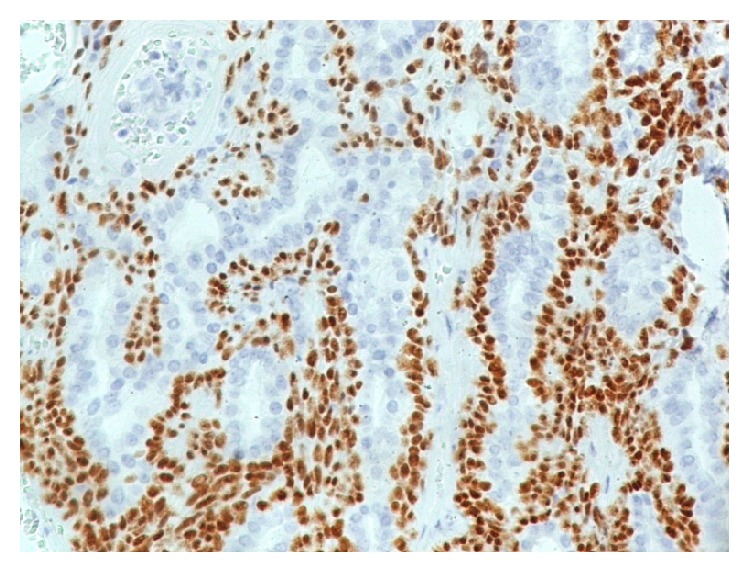
Immunostain for p63 protein (×100): nuclear positivity is seen in basal cells.

The EAC lesion was surgically excised under general anesthesia, utilizing a transmeatal approach. Macroscopically the resection specimen was a fusiform skin sample measuring 1.9 × 1.5 × 1 cm. Upon sectioning a 1.4 cm cystic lesion was found in the subcutaneous tissue. Microscopic examination showed an epithelial cystic lesion whose wall contained two cell populations. The inner ones consisted of middle-sized cylindrical or cuboidal cells with an eosinophilic cytoplasm and round nuclei, arranged in tubules (Figure 2). The cylindrical cells occasionally showed cytoplasmic projections in the lumens of the tubules. The second cell population consisted of ovoid or spindle cells, with elongated nuclei, which were arranged in fascicles, small solid groups, and rare tubules. Neither atypia nor mitotic activity were observed. The tumor was embedded in a fibrous, focally hyalinized stroma; it was partly surrounded by a thin fibrous capsule and was not connected to the overlying epidermis. Immunohistochemistry confirmed the presence of two distinct cell populations. The luminal cells expressed keratin 7, while peripheral (basal) cells expressed keratins 5/6, S100 protein, and p63. Several cells, mainly luminal, showed membranous expression of CD117/c-kit. p53 and Ki-67 were expressed in about 60% and 5% of nuclei, respectively. The apocrine gland-related antigen GCDFP-15 was focally expressed by tumor cells. Based on these histopathological and immunohistochemical findings the diagnosis of ceruminous gland adenoma was established (Figures 3 [Fig fig4]–5).

The postoperative course was uneventful and the excision site was well healed. Moreover, the symptoms of otitis media and cervical lymphadenopathy improved considerably. A week later, the ear was free of secretions and the otalgia disappeared. After 15 days, the rupture of the tympanic membrane had healed and hearing returned to its previous condition. It is possible that, due to her age and poor general condition, this patient presented with multifocal infections, such as chronic otitis media, parotitis, and cervical lymphadenopathy, which were rather coincidental to the presence of CA.

No recurrence of the CA has been noted after a 2-year follow-up.

## 3. Discussion

According to Mills et al. [1], CA are benign tumors that are rare in humans. They usually affect patients over 50 years old [1, 2, 4–6], but cases affecting adolescents exist [9]. However, the view has been expressed that all ceruminous gland tumors should be considered potentially malignant, as their clinical behavior is not yet completely elucidated [10, 11].

Ceruminous adenomas appear as reddish polypoid masses with either a smooth or ulcerated surface, which can mimic a boil [1, 2, 4, 5, 12]. Although CA produce very few symptoms, clinical manifestations such as hearing loss, mild-to-moderate otalgia, and otorrhea have been reported in some patients [2]. In our patient, the CA presented as a smooth round mass discharging a yellowish fluid upon pressure; the other symptoms such as otalgia, otorrhea, fever, and hearing loss were rather attributed to the concomitant chronic otitis media.

Ceruminous adenoma shows immunohistochemical features similar to those of normal cerumen glands, supporting their origin from these glands [1, 2]. The differential diagnosis includes other tumors that develop in the EAC, namely, exostosis, osteoma, eosinophilic granuloma, cholesteatoma, cartilaginous choristoma, extra-adrenal paraganglioma, congenital cysts of branchial arch origin, ceruminous adenocarcinoma, pleomorphic adenoma, neuroendocrine adenoma of the middle ear, and meningioma, and relies on careful immunohistological examination [2]. The recommended treatment is surgical excision of the lesion within free margins, which can ensure tumor-free survival in the long term [1, 2, 4, 5, 12]. There is no evidence of recurrence for these tumors in the literature, with a mean follow-up time of 15 years [1, 2, 4, 5, 12]. Our patient underwent total surgical excision of the mass and showed no signs of recurrence up to 2 years later.
